# Toward the design of a tailored training course for birth assistance: an Ethiopian experience

**DOI:** 10.11604/pamj.2024.47.32.42221

**Published:** 2024-01-26

**Authors:** Sabina Maglio, Selamawit Tamirat, Moges Tesfaye, Melaku Wolde, Selene Tognarelli, Arianna Menciassi, Enzo Facci

**Affiliations:** 1 The BioRobotics Institute, Scuola Superiore Sant´Anna, Pisa, Italy,; 2 Department of Excellence in Robotics and AI, Scuola Superiore Sant´Anna, Pisa, Italy,; 3 St. Luke Catholic Hospital and College of Nursing and Midwifery, Wolisso, Ethiopia,; 4 University College for Aspiring Missionary Doctors (CUAMM), Doctors with Africa, Padova, Italy

**Keywords:** Simulation, medical training, obstetrics, nursing

## Abstract

Simulation in healthcare has already demonstrated extraordinary potential in high-income countries. However, to date, few authors have explored the possibility of applying simulation-based training in African settings, highlighting the necessity of need-based training protocols capable of addressing economic, social, and cultural aspects. In this framework, this research investigates the main features of a simulation training course on umbilical cord care and placenta management should be considered effective and sustainable in an African healthcare environment. Local facilitators were identified as the best resources for defining course contents and providing technical lectures to mitigate cultural, linguistic, and social issues. For the training program, the design of a new low-cost medium-fidelity simulator was explored and a preliminary evaluation was performed. Finally, the propensity of 25 students to attend a simulation training course was investigated using a questionnaire. The attitude of the enrolled students was positive, endorsing the future introduction of simulation training into the educational offers of Ethiopian colleges.

## Introduction

Simulation in healthcare links classroom learning and real-life clinical experiences [1,2]. Patient safety, error prevention, replicable cases, confidence-building, and team training are the most recognized benefits of simulation [3]. In the past decade, some authors have questioned whether there is room for medical simulation in the African healthcare system [1-6], whereas others have implemented simulation experiences with medical students or trainees [7-9] The establishment of a simulation center in Rwanda has also been documented [10]. These data demonstrate a concrete opportunity for medical simulation in Africa. The participants were engaged and satisfied with the training, although some limitations were identified. Financial and personnel shortages, as well as technical issues such as limited power and internet access, have hindered the development and spread of health simulation. To address these limitations, methodologies that actively involve local resources and technologies to ensure training sustainability are required [6]. Moreover, effective training must be patient-centered and realistic [5]; thus, socio-cultural factors and their influence on learning and teaching must be considered [4].

Following these clear literature indications, this study was designed to create a need-based training course on umbilical cord care and placenta management during childbirth. This specific topic allows the merging of the experiences gained by engineers in high-income countries´ hospitals and the local needs of African clinicians. The study was conducted at St. Luke Catholic Hospital and College of Nursing and Midwifery (Wolisso, Ethiopia) involving both permanent staff and students. The presence of students also allows us to investigate the propensity to include these specific training activities in future education plans. Developing an effective need-based training program in Africa entails identifying the most critical elements to guarantee an affordable and sustainable course. As previously reported, the literature has shown successful results with low-fidelity manikins in some African centers [3-5,10] identifying low-cost, high-fidelity models as the most promising technical solution [5]. Thus, the main aims of this study were as follows: i) define course contents according to local guidelines and resources; ii) find or realize a low-cost, high-fidelity, and sustainable manikin; iii) define teaching and administration modalities tailored to local experiences; iv) examine St. Luke College students' inclination to use physical manikin simulation training.

## Methods

The course contents and teaching modalities were defined through regular online meetings between the clinicians and engineering team at Scuola Superiore Sant´Anna (Pisa, Italy). Literature and market analysis showed some training manikins potentially adequate for umbilical cord management. However, some are expensive and clinically incomplete, whereas others are cheaper but still incomplete from an operative point of view. Therefore, by exploiting our experience in the field of high-fidelity simulators for healthcare, a dedicated simulator of the human umbilical cord and placenta was created using rapid prototyping techniques and silicone materials. The medical team and 25 third-year St. Luke College students were asked to perform umbilical cord ligation, cutting, and cleaning, and to examine simulator fidelity and usability for placental lobes, vessels, and membranes assessment tasks. Their feedback was noted and reorganized in a report revised and approved by the clinical team. Finally, the attitude of the St. Luke College students to attend a complete simulation-based training course with the simulator was assessed with an anonymous online questionnaire created by Google Forms (Google Inc., USA). No sensitive information was provided on this form.

## Results

Daily umbilical cord and placental care procedures used at St. Luke Hospital - in agreement with the Ethiopian guidelines - were chosen as reference for the training course contents. St. Luke teachers were identified as facilitators to ensure course alignment with local teaching methods and to overcome cultural and linguistic barriers. The course was held on February 6, 2023, at St. Luke spaces to ensure student comfort and long-term sustainability.

**Figure 1:**
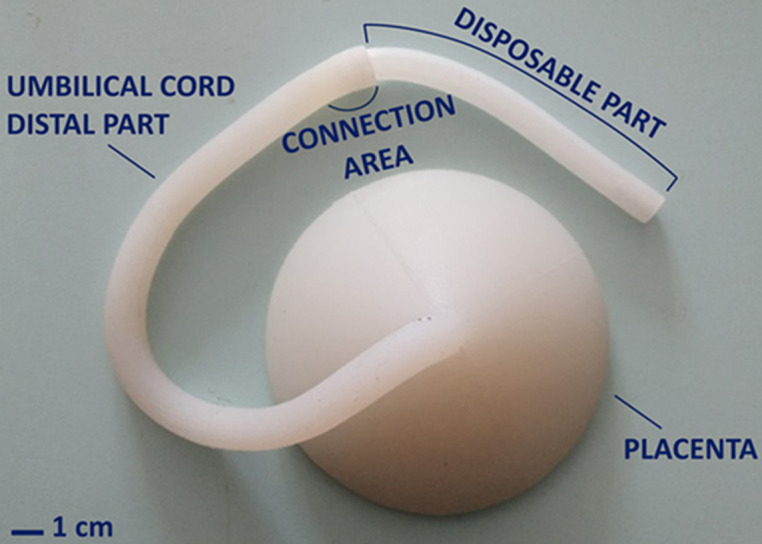
new simulator of the umbilical cord and placenta, made of silicone, with labels pointing to the main components

The new simulator (Figure 1) consisted of a reusable part and a disposable part connected through a 20 mm deep cylindrical hole exploiting the friction between the two silicone surfaces. The disposable part, a 150 mm long and 13 mm diameter cylinder, mimics the proximal part of the umbilical cord with three cylindrical holes resembling the two arteries (3 mm diameter) and one vein (3.5 mm diameter). This part is cut during the training and, due to design constraints, it can be used no more than 3-5 times, then it must be replaced. The reusable part was a 320 mm long, 15 mm diameter cylinder with a conical end 55 mm tall and 130 mm maximum diameter. It replicates the distal part of the umbilical cord and placenta and can be used repeatedly. The components were made of uncolored Ecoflex 0010 silicone (Smooth-On, USA) using molding techniques. This inexpensive silicone exhibits a balance between softness and integrity, thereby mimicking the tactile feedback of human tissues. The molds, designed using Fusion 360 CAD software (Autodesk, USA), were 3D printed using a Prusa MK3S+ printer (Prusa Research, Czech Republic) with PLA (RS Components, UK).

During simulator use, the limitations due to the absence of crucial anatomical structures, i.e. the placenta lobes and membranes, have appeared as evident, and the physiological tissue colors necessary for teaching. The material softness and possibility of being cut were appreciated. However, the high stretchability of silicone has been identified as a problem. The questionnaire was completed by 25 students, 12 nurses, and 13 midwives, and the results are presented in Table 1. Overall, the students had a positive attitude. The 24% were unaware of simulation-based education. All participants enjoyed the simulator despite its limitations and wanted to include the simulation in their study plan. They felt confident using the simulator for future re-training. 96% of the students recognized the simulation´s potential to improve their skills and confidence.

**Table 1:** list of questions provided to 25 students using the questionnaire; the results are shown as percentages

Question	Results
Did you know what the training with simulation was before today?	24%; no
	76%; yes
What are your feelings regarding simulation?	100%; positive
	0%; negative
	0%; neutral
Did you like the experience with the new simulator?	100%; yes
	0%; no
Do you think simulation can help you improve your skills?	96% yes
	4%; maybe, not sure
	0%; no
Do you think simulation can help you improve your confidence?	96%; yes
	4%; maybe, not sure
	0%; no
Would you like to introduce the simulation into your curriculum?	100%; yes
	0%; no
Would you like to have periodical training sessions on umbilical cord management?	96%; yes
	4%; no
Would you be comfortable using the simulator on your own for future individual retraining?	100%; yes
	0%; no
Would you like to participate in additional training sessions with other simulators to enhance your proficiency in other areas?	100%; yes
	0%; no

## Discussion

**Figure 2:**
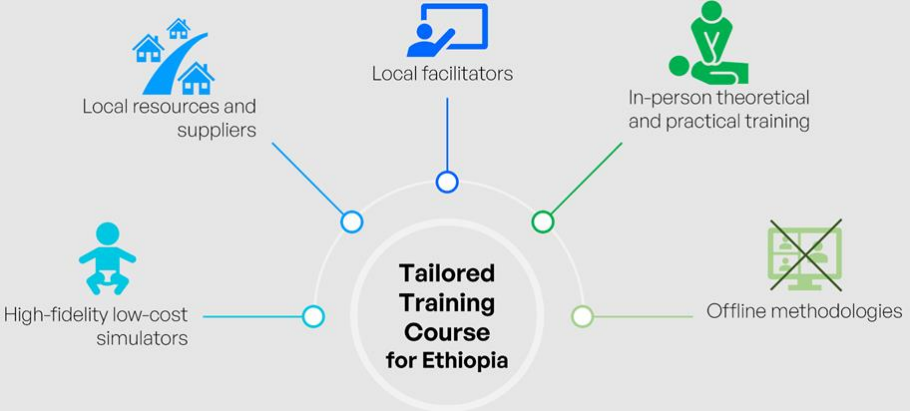
summary of key research findings; main features a simulation training course tailored to the Ethiopian needs should present

The key findings of this study are summarized in Figure 2. The research results suggest that local facilitators should define course content and teaching methods to ensure socio-cultural-linguistic compliance. Internet discussions between the two teams, clinical and engineering, experienced connectivity issues, with challenges in mutual comprehension. These issues were promptly resolved during an in-person meeting before the course. Similar connection difficulties arose during the online survey, which were eventually solved by local teachers. It is clear that Internet connectivity in Africa is still unreliable; therefore, printed surveys and in-person meetings should be preferred whenever possible.

Regarding language barriers, students were able to read English sentences, but they had difficulties speaking. Local facilitators helped address this issue. It is crucial that each participant fully understands the concepts taught because incomplete or misinterpreted information may have serious consequences for patient safety in future clinical practice. In conclusion, working with local teachers is mandatory to provide an effective tailored training course because a foreign facilitator would not be able to properly address local language, cultural, and social factors. If local teachers have no prior experience with simulation, it is recommended to 'train the trainers' before delivering the course, as demonstrated in [9].

The simulator material and manufacturing choices were selected to keep the final cost low, resulting in a medium-fidelity solution for umbilical cord cutting and basic umbilical cord care training. Owing to the limited fidelity of the simulator, healthcare students cannot learn all the steps of placenta management and umbilical cord care, such as evaluating the right color of umbilical cord structures and identifying placental components. The silicone tactile feedback in compression resembles human tissues, but its high stretchability does not replicate reality. The real umbilical cord is inextensible and usually pulled to help deliver the placenta, which cannot be achieved using this simulator. Thus, improvements in anatomical and appearance fidelity are required. Finally, silicone, which is affordable in wealthy countries, is difficult to obtain in Ethiopia. At the same time, disposable part delivery is costly and unsustainable. Therefore, to create a locally manufactured and sustainable product, the identification of another material for the simulator is required. Further design choices should target a compromise between fidelity, affordability, and local manufacturability to lean toward the low-cost high-fidelity sustainable models demanded, e.g. by Campain *et al*. [5].

## Conclusion

Literature shows that there is potential for the use of medical simulations in Africa, provided that local socio-cultural-economic factors are considered. The design of a sustainable and need-based simulation training course was investigated. Procedural guidelines and best teaching and administration practices were identified. A new medium-fidelity low-cost simulator was developed and assessed by the local clinical team and students. It showed a few strong limitations that require further improvement before being used in a training course. Students showed a generally positive behavior toward simulation, demonstrating that there is space for simulation training in the educational offer of Ethiopian colleges.

### What is known about this topic

- *The utilization of simulation as a teaching tool in medical education is considered to be effective in African nations;*
- *It is necessary to employ methodologies that actively engage local resources and technologies to ensure the sustainability of training*.

### What this study adds

- *Guidelines for structuring a tailored training course for African medical students are provided;*
- *It is recommended that low-cost, high-fidelity simulators be utilized for effective training courses in low-income countries*.
